# Voriconazole-Induced Cholestatic Hepatotoxicity in an Immune Competent Patient

**DOI:** 10.7759/cureus.21346

**Published:** 2022-01-17

**Authors:** Yaser Mohammed, Ahmed Abousamra, Ahmed Atef I Abdeldayem, Mansoor Zafar, Tila Muhammad

**Affiliations:** 1 Internal Medicine, Conquest Hospital, East Sussex Healthcare NHS Trust, St Leonards-on-Sea, GBR; 2 Intensive Care Unit, Conquest Hospital, East Sussex Healthcare NHS Trust, St Leonards-on-Sea, GBR; 3 Gastroenterology and Hepatology, Conquest Hospital, East Sussex Healthcare NHS Trust, St Leonards-on-Sea, GBR

**Keywords:** deranged liver function tests, cholestatic liver injury, dose titration, invasive aspergillosis, voriconazole toxicity

## Abstract

Voriconazole is a frequently prescribed anti-fungal medication used in particular for invasive aspergillosis. Voriconazole-induced hepatotoxicity is a relatively rare but serious clinicopathologic entity. We report a patient presenting with impaired liver function test results pointing towards the cholestatic picture. The patient had initial blood tests to confirm cholestasis, followed by imaging modalities that did not show any obstruction along the common bile duct and/or pancreatic pathway. The patient’s voriconazole dose reduction was advised, resulting in lower levels of abnormal liver function tests.

## Introduction

Voriconazole is a clinical challenge for patients with invasive aspergillosis, as the drug level varies between each patient towards therapeutic index. Additionally, in the same patient the levels may vary. The literature mentions cholestatic picture for lab results, as a consequence of being on voriconazole in patient's post-liver or renal transplant, chronic liver disease, or any immunocompromised patient [1].

However, there is scant information about the cholestatic picture for a patient managed with voriconazole who is immune competent, which makes this case unique in the sense of being mindful of the risks put forth by voriconazole and that dose titrations need be appropriate [2-4].

## Case presentation

**Figure 1:**
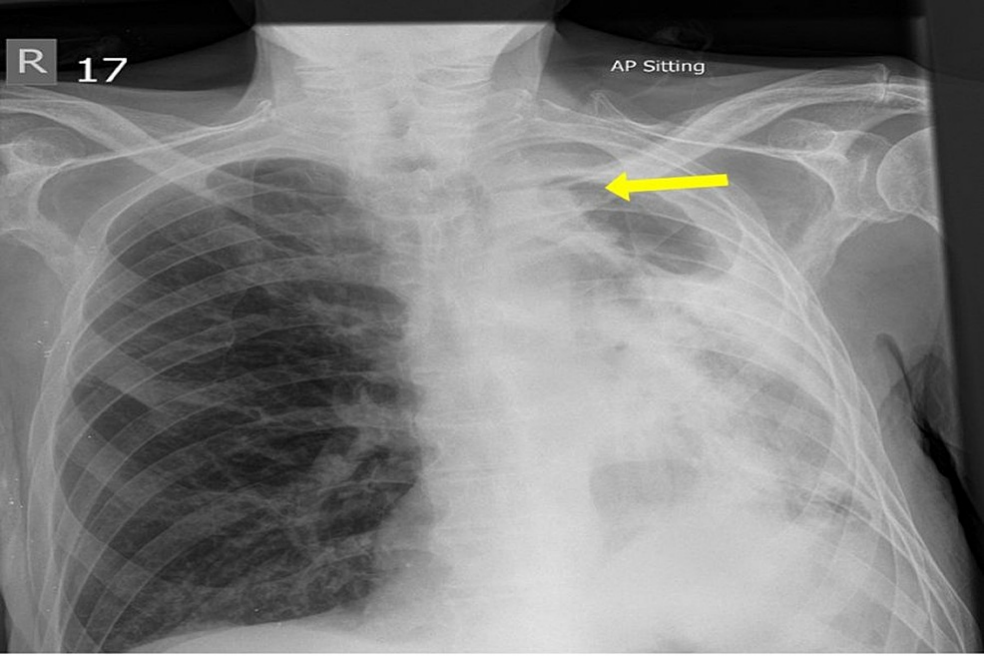
Chest X-ray: Antero-posterior view. There are long-standing changes, loss of left lung volume, left apical cavitation (yellow arrow), and extensive pleural thickening together with chronic fibrotic changes within the left upper lobe.

**Figure 2:**
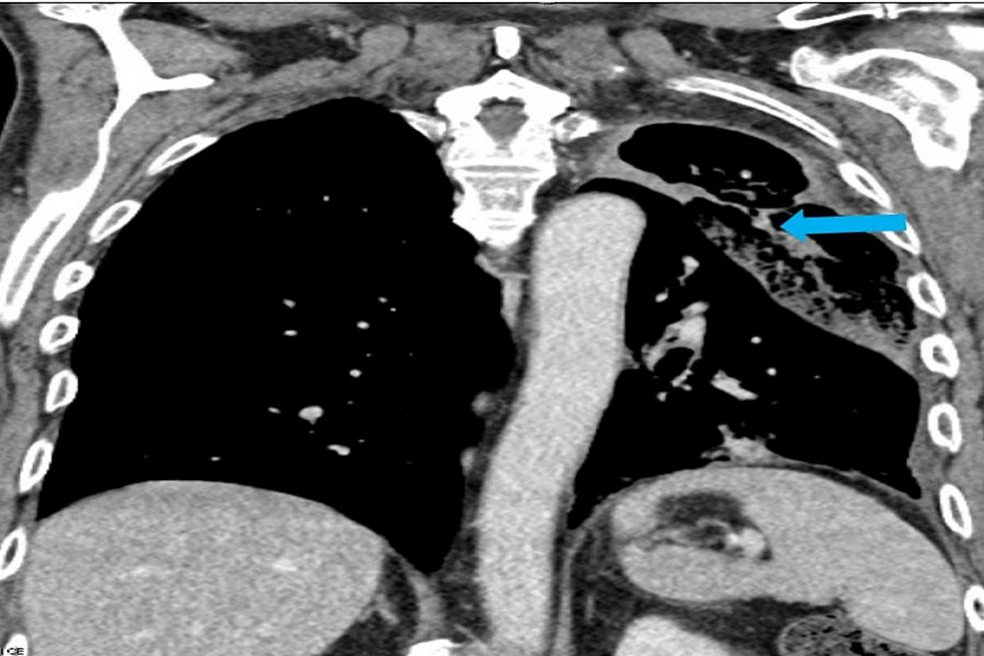
Computed tomogram (CT) Chest- Coronal view. Marked volume loss in the left upper lobe with a large cavity containing some soft tissue density material the appearances of which are those of an aspergilloma (blue arrow).

A 71-year-old male, resident of a nursing home, called the ambulance to come to the hospital for worsening fatigue, tiredness, generalized weakness. He had a background history of severe frailty, a history of basal cell carcinoma skin excised from left temple with complete resolution, and on voriconazole treatment of 300 mg twice daily (12 hourly) for invasive aspergillosis (Figures 1, 2).

Previously while under the respiratory team his case was discussed with the thoracic surgery team at a tertiary centre for lobectomy and deemed unfit for surgery for severe frailty. During the current admission he was reviewed by the gastroenterology team during ward rounds with no abnormalities detected on clinical examination. Following his blood test results were analysed. They revealed deranged blood liver function tests (LFTs) with alkaline phosphatase 362 (normal range 30 - 130 IU/l), alanine transaminase (ALT) 93 (normal range 5 - 30 IU/l) (Table 1), C-reactive protein (CRP) 106 (normal range 0-5), white cell count (WCC) 9.32 (normal range 4-11 x109/litre), international normalization ratio (INR) 1, and platelets 541 (normal range 150-400 x 109/litre). His Aspergillus immunoglobulin IgG level in the last three months was persistently >200 while immunoglobulin IgE was 0.01.

**Table 1:** Laboratory parameters with down-trend of Liver Function Tests. *Therapeutic Trough Level.

Serum markers	units	Normal range	Day1	Day 3	Day 10	Day 20
Serum Bilirubin (total)	umol/L	0 - 21	14	11	10	10
Serum Alkaline Phosphatase (ALP)	U/L	30 - 130	362	353	304	262
Serum Alanine Transaminase (ALT)	U/L	10 - 35	93	73	62	30
Serum Voriconazole level	mg/L	1-5.5^*^	7	6.5	3	2
Serum Albumin	g/L	35 - 50	20	21	22	30

He was found to be on high-dose voriconazole treatment with no history of being immune compromised, and no history of chronic liver disease. His non-invasive liver screen blood tests, including tuberculosis screening tests were completely negative. He underwent an ultrasound that was inconclusive with a normal liver echo-texture. Magnetic resonance cholangiogram (MRCP) was performed and suggested few gallbladder stones, and despite movement artefacts, his common bile duct (CBD) was not dilated. No pancreatic and/or CBD calculi were visualized. Gall bladder though with stones, did not demonstrate any inflammatory changes or sinister pathology. It was postulated that voriconazole is the reason for the abnormal LFTs via cytochrome P450 enzyme induction. A downward trend of his LFTs was noted with dosage reduction of the voriconazole medication to 200 mg twice daily with a faster improvement in symptoms of fatigability and tiredness. The patient was deemed medically stable and discharged back to the nursing home, with an advice to the general practitioner (GP) for a periodic fortnightly blood liver function tests to ensure a downtrend towards normalization. He was reviewed six months later in an out-patient gastroenterology clinic with normalization of the blood liver function tests.

## Discussion

The most recent case reports published in 2011, 2013, 2014, 2015, and 2019 that mentioned cholestatic liver function test results with voriconazole use reported these findings in immunocompromised patients [5,6]. Immunocompromised patients are prone to have fungal or disseminated fungal infections and are managed with anti-fungal medications like voriconazole. It is important to monitor any complications of anti-fungal therapy [7,8]. 

Immune competent patients may develop fungal infections, although less frequent than immunocompromised, and are still prone towards side effects of anti-fungal medications such as cholestatic LFTs. Appropriate steps are needed to be taken towards dose titration or change to alternate medications under the supervision of an infectious disease specialty physician [9,10]. 

It is recommended that in patients aged ≥12 years, intravenous dosing consists of 6 mg/kg twice daily on day one followed by 4 mg/kg twice daily for the remainder of treatment. Oral dosing in adults is weight based. Adult patients weighing >40 kg should receive 400 mg twice daily on day one followed by 200 mg twice daily until the end of therapy. A 200 mg twice-daily loading dose on day one followed by 100 mg twice daily, is recommended for those weighing <40 kg. Clinicians prefer oral dosing to intravenous dosing by administering the loading dose of 6 mg/kg and maintenance dose of 4 mg/kg doses rounded up to the nearest pill size (oral formulation available as 200 mg and 50 mg tablets). For the paediatric population dosing reflects the rapid metabolism and linear kinetics with a recommendation of 7 mg/kg twice daily intravenously and 200 mg orally twice daily, however, without loading doses [11, 12].

The British National Formulary (BNF) under National Institute for Health and Care Excellence (NICE) in the United Kingdom (UK) advises initially 200 mg every 12 hourly for two doses, then 100 mg every 12 hourly, increased if necessary to 150 mg every 12 hourly for adults with body weight up to 40 kg. However, initially 400 mg every 12 hourly for two doses, then 200 mg every 12 hourly, increased if necessary to 300 mg every 12 hourly is recommended for adults with body weight 40 kg and above [13]. The trough levels recommended should be maintained between 1 - 5.5 mg/L. If levels are found to be lower, appropriate dosing needs to be ensured in relation to meals if given orally, while also considering increasing the dose and examining the effects of concomitant medications [14]. 

Although literature points towards increased association of cholestasis with deranged LFTs in immunocompromised patients on voriconazole, we attempted to highlight similar cholestasis in association with voriconazole in immune competent patient. It remains important to consider medication-associated cholestasis, with resolution of symptoms after dose titration. 

## Conclusions

Immune competent patients similar to immunocompromised patients may develop fungal infections, although less frequent than immunocompromised, and are still prone to side effects of anti-fungal medications such as cholestatic liver injury with deranged LFTs. Appropriate steps are needed to be taken towards dose titration or change in medications under the supervision of an infectious disease specialty physician.
